# Malaria and missed school days: exploring school absenteeism patterns and local strategies in Odisha, India

**DOI:** 10.3389/fpubh.2025.1502247

**Published:** 2025-02-27

**Authors:** Muhammed Jabir, Dilip K. Panigrahi, Muhammad M. Baig, Vijayakumar Balakrishnan, Prasant K. Panda, Ashwani Kumar, Manju Rahi, Ananganallur N. Shriram

**Affiliations:** ^1^Department of Epidemiology and Operational Research, ICMR- Vector Control Research Centre, Puducherry, India; ^2^ICMR-Vector Control Research Centre, Field Station, Koraput, Odisha, India; ^3^Department of Biostatistics and VBD Modelling, ICMR- Vector Control Research Centre, Puducherry, India; ^4^Department of Economics, Pondicherry University, Puducherry, India; ^5^Saveetha Institute of Medical and Technical Sciences, Saveetha University, Kanchipuram, Tamil Nadu, India; ^6^ICMR-Vector Control Research Centre, Puducherry, India; ^7^Division of Vector Biology and Control, ICMR-Vector Control Research Centre, Puducherry, India

**Keywords:** malaria, school absenteeism, children, education, India, Odisha, local strategies

## Abstract

**Background:**

Malaria continues to pose a significant global health challenge, disproportionately affecting children. While its effects on physical health are well-documented, the impact on education, particularly school absenteeism, remains less understood. This study aimed to explore the influence of malaria on school absenteeism in Odisha, India.

**Methods:**

A mixed-methods study was conducted in four southern districts of Odisha from September 2023 to February 2024. This involved a retrospective analysis of school attendance registers from five primary schools and qualitative interviews with 25 school teachers. Statistical analysis was performed using SATA 14.1. Mixed effects logistic regression analysis was used to determine the predictive factors of absenteeism with independent variables such as year, area of school and class. Qualitative data from interviews were transcribed and thematically analyzed.

**Results:**

The study evaluated absenteeism among 832 children from Class 1 to Class 5 across four academic years (2016–2020), with schools averaging 185 working days annually. While absenteeism rates varied by school, the proportion of students with yearly absenteeism rates of ≥30% remained relatively stable, ranging from 6 to 12.1%. Average absenteeism ranged from 11.5 days in the academic year 2016–2017 to a peak of 22.6 days in 2018–2019. Logistic regression analysis revealed no significant association between malaria endemicity and absenteeism patterns. Schools employed several malaria prevention and case management strategies, including health education, long-lasting insecticidal nets (LLINs), vector control, screening, testing, and on-site treatment. However, resource constraints and cultural barriers continue to pose challenges.

**Conclusion:**

Despite a decline in malaria cases in the study area, school absenteeism persists due to factors beyond malaria. Future interventions should address these broader socio-cultural and logistical issues to effectively manage absenteeism and improve educational outcomes in malaria-endemic regions.

## 1 Introduction

Malaria, caused by parasites of the genus *Plasmodium*, continues to pose a significant global health challenge, with 263 million reported cases and 597,000 deaths worldwide in 2023 (1). The prevalence of malaria among school-going children is notably high (2–4), with over 500 million children estimated to be at risk globally (5). School-aged children also serve as an important reservoir for malaria transmission, fueling the spread of the disease to higher-risk groups (6). The impact of malaria on school-aged children extends beyond physical health, significantly affecting their education and overall quality of life. Research consistently highlights the adverse effects of malaria on academic performance, school attendance, behavior and cognitive development (7–9). Children suffering from malaria in deprived areas are at greater risk of school absenteeism and lower educational attainment (10). For instance, malaria is responsible for 5–8% of school absences in the African region (11, 12). Beyond its direct consequences, malaria also hampers children’s access to education by disrupting family dynamics and imposing caregiving responsibilities (13).

In recent years, substantial efforts have been made to expand malaria interventions beyond the formal health sector, especially in resource-constrained settings, to address gaps in diagnosis and treatment. Schools, as an ideal platform for malaria prevention, have become central to these efforts (3, 14) and have proven to be both feasible and effective (9). Recent meta-analyses suggest that preventive treatments in schools could reduce malaria incidence among school children and related absenteeism (15). Several studies conducted in Africa demonstrated the effect of school-based interventions, with presumptive malaria treatment administered within schools leading to improvements in attendance, academic performance and overall health outcomes (11, 13, 16, 17). Additionally, the use of mosquito nets by school-aged children has been shown to significantly reduce the risk of malaria infection (18, 19). In India, the National Strategic Plan for Malaria Elimination (NSP) 2023–2027 emphasizes integrating malaria awareness into middle-school health education programs and engaging teachers in malaria surveillance and vector control strategies (20). Previous studies in India have also highlighted the effectiveness of school-based educational interventions in enhancing knowledge of malaria prevention and control among children (21, 22).

Despite ample evidence on malaria patterns, clinical characteristics, vectors’ behavior, and effects of control strategies (23–25), much less is known about the effect of malaria on school absenteeism in India. It remains unclear whether shifts in malaria trends over the years have any impact on school absenteeism in endemic communities. Thus, this study aims to explore this aspect in an endemic setting in Odisha, a historically high malaria burden area where the incidence and proportion of *P. falciparum* species has been very high. The study also aims to understand the local malaria prevention strategies by schools in endemic areas, as well as the ongoing challenges they face. As likely the first study of its kind in India, it seeks to provide valuable insights into malaria and school absenteeism, contributing to both public health discourse and policy development.

## 2 Materials and methods

### 2.1 Study setting

**Figure 1:**
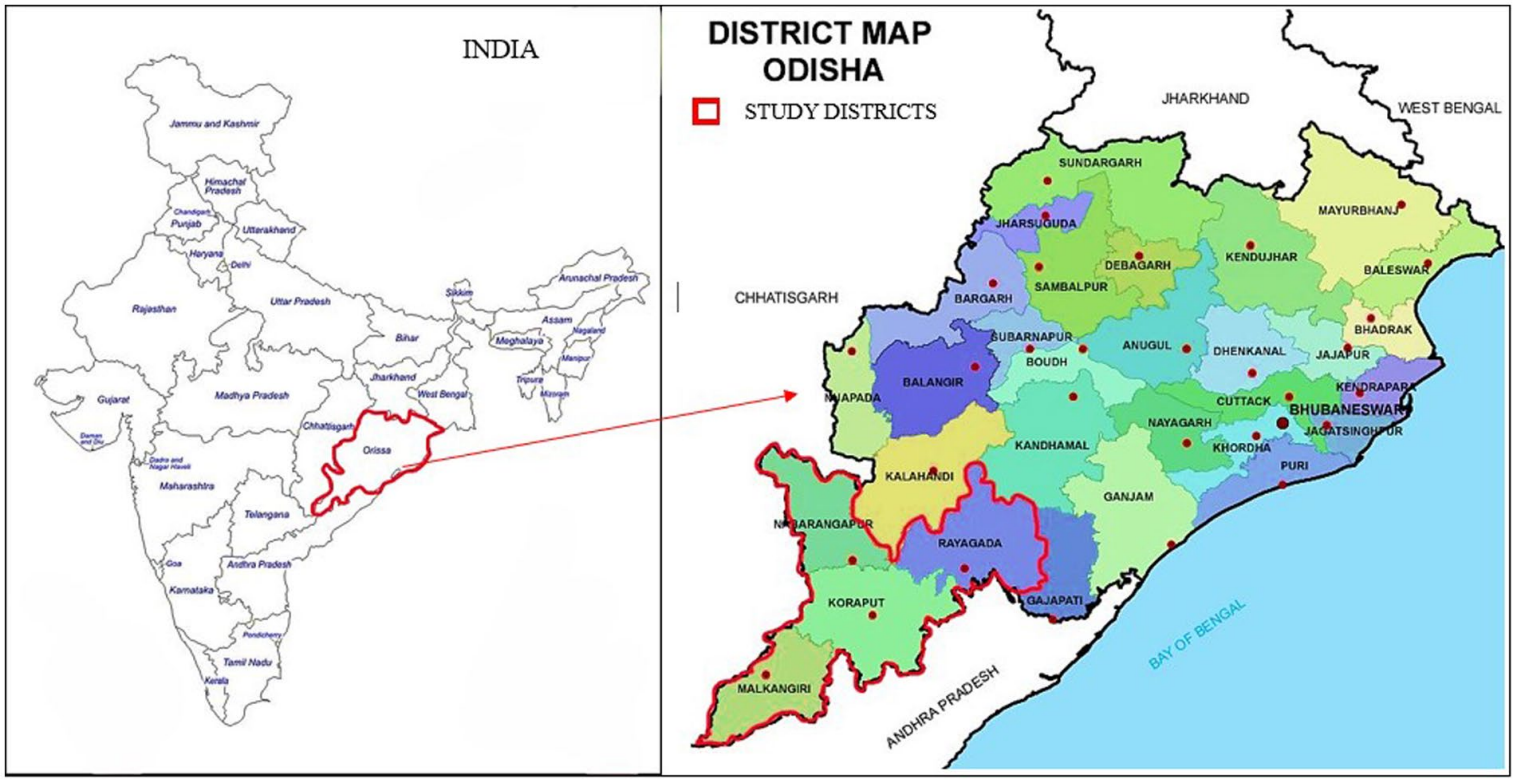
Map of Odisha showing the four study districts (red border).

This study was conducted in four southern districts of Odisha state in India. The districts include: Rayagada, Nabarangpur, Malkangiri, and Koraput, which are severely endemic for *falciparum* malaria (Figure 1). These districts share common characteristics, including dense forest cover, and a prolonged history of malaria. The inhabitants of the study area are predominantly indigenous tribal communities. Access to educational facilities is limited, with residents heavily reliant on government services.

### 2.2 Study design

**Figure 2:**
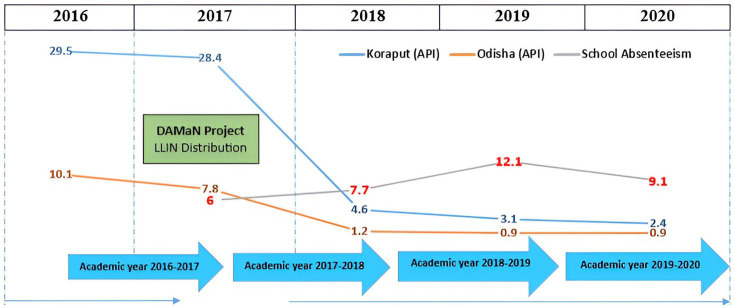
Chronogram on academic years, malaria API and control strategy.

We employed a sequential explanatory mixed-method research approach. Initially, we collected attendance registers of school children from five government-operated primary schools in the Koraput district, spanning the academic years 2016–2017 to 2020–2021. Most children in the study area attend government schools. The Koraput district recorded a significant decline in malaria annual parasite incidence (API: malaria cases/1000 population) from 29.5 in 2016 to 2.4 in 2020 (Figure 2). Consequently, periods of both high (2016) and low endemicity (2020) were selected to compare trends in school absenteeism. In 2017, the government of Odisha launched the DAMaN project, distributing 11.3 million long-lasting insecticidal nets (LLINs) across 23 districts in the state using incidence for prioritization for net distribution. This initiative contributed to the drastic reduction of malaria in the subsequent years.

Furthermore, we conducted a qualitative exploratory study involving in-depth interviews with school teachers to gather their perceptions on malaria and its linkage with school absenteeism. A total of 25 teachers from 22 schools across the four study districts participated in these interviews. The participants included head teachers, senior teachers and hostel wardens.

### 2.3 Tool and data collection

**Figure 3:**
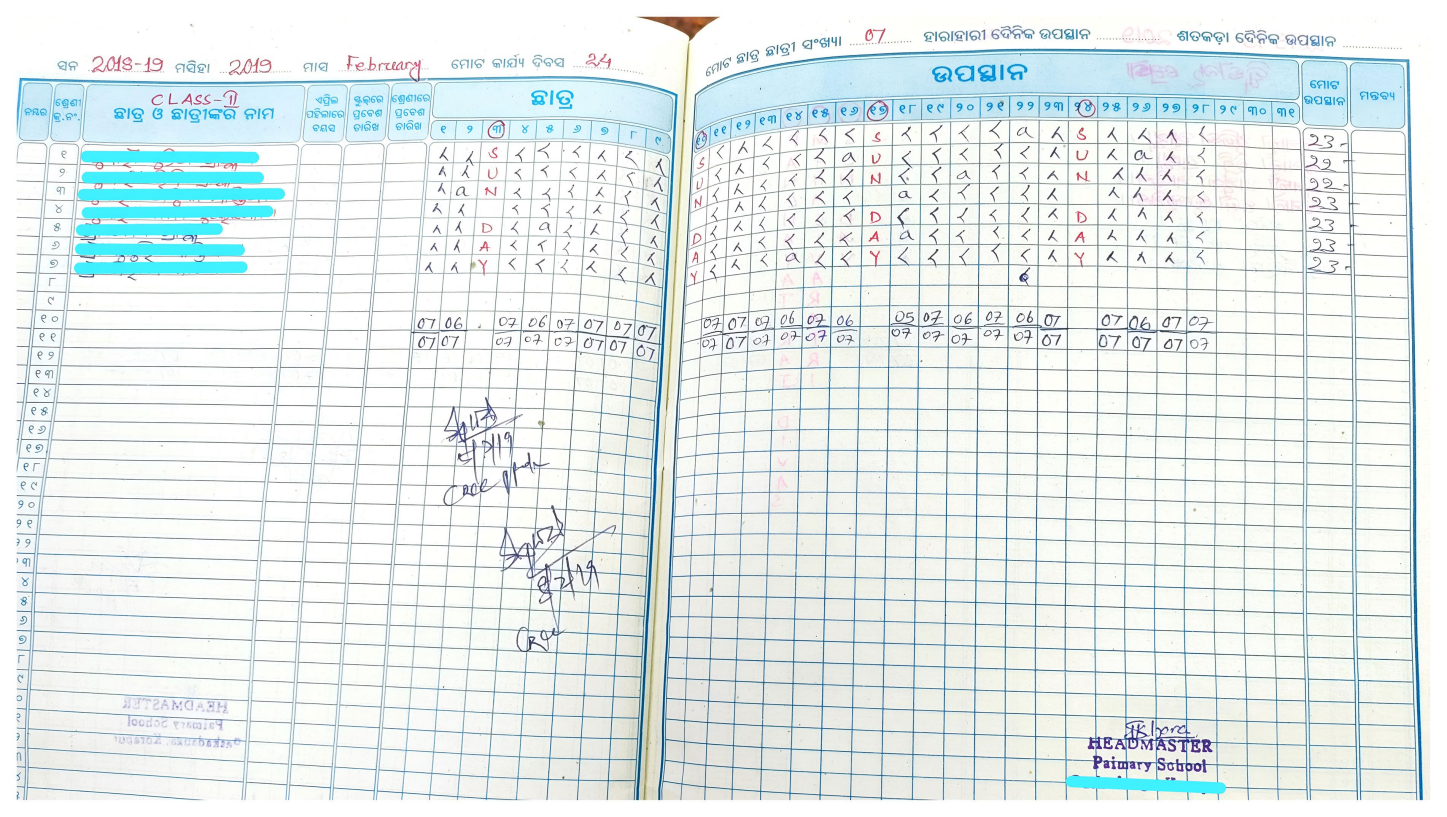
School attendance register on daily absenteeism.

Data collection was done between September 2023 and February 2024. Attendance registers were gathered by visiting each school included in the study (Figure 3). To digitize the attendance data, photos were taken of each page from the paper registries with permission from the school. These registers included daily attendance and absenteeism details of students, including student names, academic year, month, and class. In the qualitative phase, face-to-face interviews were conducted with teachers who volunteered for the study. An interview guide was used to collect demographic details and their perceptions of malaria and school absenteeism. The guide was refined through pretesting and field experiences. The interviews took place on school premises, each lasting 45–60 min. A sociologist trained in conducting qualitative interviews, supported by field workers fluent in “Odia” (local language), conducted the data collection.

### 2.4 Data analysis

Attendance data were entered into MS Excel and analyzed using STATA 14.1 (Texas, United States). The absenteeism percentage was calculated by using the formula: (Total number of days attended school/total number of working days) × 100. In India, no official metric for sickness or chronic absenteeism exists, but some researchers define absenteeism as 10–15% annually (26, 27). For this study, absenteeism was defined as students absent for more than 30% of the total working days in a year, considering the local context of socio-economic challenges and inaccessibility. Absenteeism data were presented as numbers with percentages. The chi-square test was used to determine the association of categorical variables with absenteeism. Mixed effects logistic regression analysis was used to determine the predictive factors of absenteeism with independent variables such as year, area of school and class. These variables were included to account for various factors influencing absenteeism, such as changes in malaria prevalence over time (with higher API rates in 2016 and a significant decrease by 2020), differences in local socio-economic conditions and healthcare access that could impact absenteeism rates across school settings, and variations in absenteeism patterns by academic level, as younger students may be more susceptible to illness. In this study, a *p*-value of less than 5% was considered statistically significant.

Participant’s responses from qualitative interviews were audio-recorded with their prior consent, transcribed verbatim, and translated into English. The transcripts were then analyzed thematically using a grounded theory approach. The analysis began with “open coding” to identify patterns and relevant topics in the data. This was followed by axial coding to categorize the open codes into meaningful categories. The coding framework was developed inductively, primarily derived from the researcher’s interpretation of the data, ensuring that the themes were closely tied to the participant’s responses. Two independent researchers coded the transcriptions and any discrepancies were discussed and resolved to ensure inter-coder reliability. A clear and systematic coding framework was applied consistently across all interviews, and the iterative coding process and team discussions ensured the accuracy of the analysis.

## 3 Results

**Figure 4:**
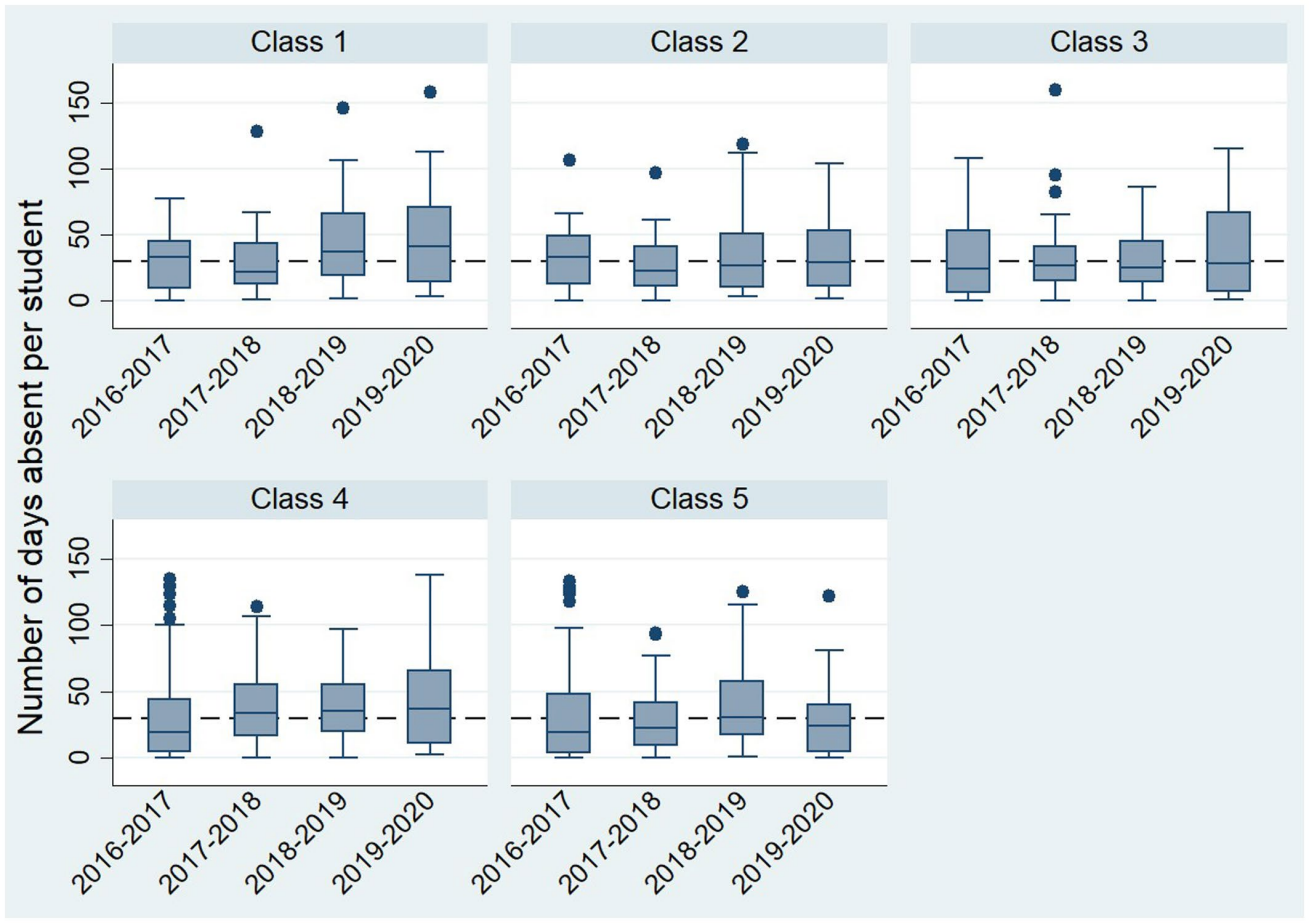
No. of days absent by class and year.

A total of 832 children from Class 1 to Class 5 were assessed in this study. The total number of working days ranged from 160 to 190. In Class 1, average absenteeism was 16 days (SD = 21.3) in the academic year 2016–2017, rising to a peak of 25.5 days (SD = 25.59) in 2018–2019. Class 2 showed an average absenteeism of 18 days (SD = 22.3) in 2016–2017, with a peak of 20 days (SD = 27.1) in 2018–2019, followed by a decrease to 13 days (SD = 12.2) in 2019–2020. For Class 3, absenteeism averaged 11 days (SD = 21.1) in 2016–2017 and significantly decreased to 7 days (SD = 22.9) in 2019–2020. Class 4 recorded the highest average absenteeism, with 24 days (SD = 22.8) in 2018–2019, compared to just 7 days (SD = 21.2) in 2016–2017. In Class 5, absenteeism averaged 5.5 days (SD = 17.2) during the academic year 2016–2017, peaking at 24 days (SD = 29) in 2018–2019 (Figure 4).

The yearly absenteeism rates of ≥30% in the study area for the academic years 2016–2017 through 2019–2020 were 6, 7.7, 12.1, and 9.1%, respectively. The absence trends over the four academic years did not show any statistically significant difference, suggesting that despite a substantial decline in malaria cases during this period, malaria alone may not have influenced absenteeism rates. Nevertheless, significant disparities in absenteeism were evident across different schools, with Balingi having the lowest rate at 1.3% and Musipalli recording the highest at 3.6%. This indicates that factors specific to each area, such as local socioeconomic conditions or school environment, might have influenced absenteeism patterns. The difference in absenteeism between areas was statistically significant (*p* < 0.001). Class-wise absenteeism rates ranged from 10.2 to 7.4% from standard 1 to 5, respectively. However, no significant difference was observed across different classes within the studied population (Table 1).

**Table 1:** School absenteeism (≥30 days) by year, area, and class.

Variable	Absenteeism		*P*-value*
	Total	n (%)	
Year			
2016–2017 2017–2018 2018–2019 2019–2020	269 220 190 153	16 (6.0) 17 (7.7) 23 (12.1) 14 (9.1)	0.126
Area			
Balingi Dhamanganda Minapai Musipalli Piskadango	151 195 251 49 186	2 (1.3) 35 (18.0) 16 (6.4) 15 (30.6) 2 (1.1)	<0.001
Class			
I (5–6 years) II (6–7 years) III (7–8 years) IV (8–9 years) V (9–10 years)	166 153 153 198 162	17 (10.2) 8 (5.2) 11 (7.2) 22 (11.1) 12 (7.4)	0.276

The ‘*’ in table indicates the use of the “chi-square test”.

Table 2 presents the results of a logistic regression analysis examining the association between school absenteeism and the variables of year, class, and their interaction. The analysis found no significant association between absenteeism and year (OR = 0.98, 95% CI: 0.57–1.67, *p* = 0.940), class (OR = 0.91, 95% CI: 0.67–1.25, *p* = 0.569), or the interaction of year and class (OR = 1.09, 95% CI: 0.92–1.29, *p* = 0.300). These results suggest that school absenteeism did not significantly differ across the academic period or between different classes in this study.

**Table 2:** Logistic regression analysis of absenteeism.

Variable	OR (95% CI)	*P*-value
Year	0.98 (0.57–1.67)	0.940
Class	0.91 (0.67–1.25)	0.569
Interaction	1.09 (0.92–1.29)	0.300

### 3.1 Results from qualitative study

This qualitative investigation aimed to engage with key stakeholders, including teachers and other relevant individuals, to elucidate the emerging trends of malaria among school children, underlying causes, disease management practices, absenteeism trends, and challenges in primary education settings in the study area.

### 3.2 Demographic characteristics

The qualitative study included a total of 25 school teacher participants, comprising 14 males and 11 females. The majority belonged to the age category of 50 and above years (*n* = 14). Most of the participants were working as head of the school (Headmaster or headmistress) (*n* = 13), followed by assistant teachers (*n* = 10) and hostel wardens (*n* = 2). A significant majority reported having more than 5 years of experience (*n* = 18) (Table 3).

**Table 3:** Characteristics of participants.

Characteristics	*n* = 25
Gender	
Male	14
Female	11
Age	
30–39	5
40–49	6
≥ 50	14
Role/Designation	
Head Teacher	13
Assistant teacher	10
Hostel warden	2
Experience in the present school	
<5 years	7
>5 years	18

### 3.3 Themes and categories from qualitative analysis

In this section, we present the major themes and categories that emerged from the qualitative analysis of in-depth interviews conducted with teacher participants. We use illustrative quotations from participants to describe and support the findings. These themes reflect the key experiences and perceptions regarding malaria, highlighting insights into its transmission among school children, control strategies, and other aspects (Table 4).

**Table 4:** Major themes and categories from patient interview.

Themes	Categories
Malaria trends	Decline
	Seasonal fluctuations
Facilitating factors	Health education
	Long lasting insecticidal nets (LLIN)
	Healthcare accessibility
	Vector control
Preventive strategies	Health education
	Hygiene and vector control
	Long lasting insecticidal nets (LLIN)
	Traditional remedies
	Health screening
	Teacher training
Responsive strategies	Diagnostic testing
	On-site treatment and referral
	Sick rooms
Malaria and school absenteeism	
Perceived challenges	Lack of trained teachers
	Inadequate supply of LLINs
	Absence of dedicated ANMs
	Traditional healing practices
	School vacation
	Migration to *kudias* (distant farm huts)
	Lack of risk perception among parents

### 3.4 Malaria trends

This section explores the landscape of malaria infection among primary school children.

#### 3.4.1 Decline

There has been a significant reduction in malaria among primary school children in the study area in recent years, as reported by respondents. They noted that fever cases, once commonly diagnosed as malaria, are now predominantly due to colds or viral fevers. Participants shared their observations:

*It (malaria) has been significantly reduced compared to previous years. The fever cases I encounter are mostly cold and viral infections, not malaria. The change has been noticed even in my hometown. (P6, F, Head Teacher)*

*I reside within this school campus. I can confirm that there have been no cases of malaria in our school for the past five to six years. (P2, F, Teacher)*

Furthermore, this decline in malaria is not confined to the school boundaries but extends to the surrounding communities and villages.

*I have not come across malaria-positive cases in our school or the surrounding areas. About six years ago, anyone with a fever in our school was typically diagnosed with malaria. (P9, F, Teacher)*

#### 3.4.2 Seasonal fluctuations

Despite improvements, fluctuations in malaria incidence were noted, with some participants reporting recent increases in cases, especially during the rainy seasons.

*I think malaria appears to be a seasonal disease. During the rainy season, we tend to see an increase in cases. Last rainy season, we found 4 to 5 malaria cases in our school. (P3, M, Teacher)*

*I have seen a slight increase in malaria cases, especially during the rainy season. However, compared to five years ago, the situation has significantly improved. Malaria is no longer as severe of a concern as it once was. (P14, M, Teacher)*

### 3.5 Facilitating factors

This section examines the participant’s perceptions regarding the factors that contributed to the reduction of malaria among primary school children.

#### 3.5.1 Health education

Participants unanimously highlighted the significant impact of government interventions in malaria control, particularly the proactive involvement of various healthcare stakeholders such as ASHA (Accredited Social Health Activist) and Anganwadi workers. They stated that the grass-root level health workers were instrumental in disseminating information and fostering behavioral changes among the community.

*I believe that it is (malaria reduction) largely due to the intensive awareness programs conducted by the government during the past many years. (P2, F, Teacher)*

*I think the awareness created by the health department, as well as by the Anganwadi workers and ASHA has helped to bring down the cases in our area. (P6, F, Head Teacher)*

*Our students are now well aware that the regular use of mosquito nets can prevent mosquito bites and malaria. (P3, M, Teacher)*

#### 3.5.2 LLINs

Long-lasting insecticidal Nets (LLINs) played a significant role in reducing malaria, especially among school children, as pointed out by respondents. The use of LLINs by children in school hostels was particularly highlighted as effective in controlling malaria.

*After they (students) started using the insecticide-treated nets, malaria cases decreased drastically. (P1, F, Hostel Warden)*

*I noticed that the use of Long-Lasting Insecticidal Nets (LLINs) by children in the hostel has reduced mosquito bites and consequently, malaria transmission. (P18, M, Head Teacher)*

#### 3.5.3 Healthcare accessibility

Improvements in healthcare facilities contributed to reducing malaria among school children, as reported by school teachers. Participants pointed out a notable improvement in healthcare accessibility in the region over the past several years. The provision of diagnostic kits and treatment within schools enabled prompt and accurate detection of malaria cases, facilitating timely treatment.

*In the past 8 to 10 years, I have seen significant improvements, mainly due to the availability of testing kits. (P 15, M, Head Teacher)*

*The availability of treatment within school premises has contributed to this improvement in malaria. (P5, F, Teacher)*

#### 3.5.4 Vector control

The concerted efforts to improve environmental hygiene by clearing mosquito breeding grounds resulted in a reduction in mosquito density and malaria.

*In the past, our hostel surroundings were full of jungle and mosquito breeding grounds, but now it is somewhat clean, which has reduced the mosquito density in our locality. (P1, F, Hostel Warden)*

### 3.6 Preventive strategies

This part explores the strategies implemented by schools to prevent malaria. The following quotations shed light on the diverse approaches adopted by them to combat malaria.

#### 3.6.1 Health education

Participants stated that students are educated about good hygiene practices and the importance of using mosquito nets to prevent malaria. They integrate malaria preventive messages into prayer classes, leveraging the platform for behavioral change among students. The efforts extend beyond the school grounds, as evidenced by organizing rallies in the villages to educate residents about malaria.

*We educate students about good hygiene practices and emphasize regular use of mosquito nets. (P2, F, Teacher)*

*We incorporate messages about malaria prevention into our prayer classes and organize rallies in the village to educate residents about malaria and its control. (P8, M, Teacher)*

#### 3.6.2 Hygiene and vector control

The participants provided insights into measures taken to mitigate mosquito breeding and ensure a hygienic environment within the school campus. The hostel warden emphasized the importance of clearing bushes near the hostel premises to eliminate mosquito resting sites. They highlighted the regular use of bleaching powder in overhead tanks and the maintenance of cleanliness throughout the campus, particularly during the rainy season. Additionally, one of the participants mentioned that indoor residual spraying is done as part of routine measures to control mosquito breeding.

*We clear bushes near the hostel to eliminate mosquito resting sites. (P1, F, Hostel Warden)*

*We regularly use bleaching powder in overhead tanks and maintain cleanliness in the campus. During the rainy season, we clean drains and waterlogged areas. (P24, M, Teacher)*

*The government conducts indoor residual spraying regularly to control mosquitoes. (P 11, M, Head Teacher)*

#### 3.6.3 LLINs

Another key preventive strategy is the provision of mosquito nets to students, especially for hostel dwellers. Most of them reported the use of mosquito nets, with meticulous monitoring systems in place to ensure compliance among students. The hostel warden monitors the correct tying of nets by each child in the hostel, which enhances the efficacy of the nets.

*We monitor mosquito net usage during sleeping hours to ensure all students are protected. We even check the proper tucking of the net by each child in our hostel (P4, M, Teacher)*

*We stress regular net usage among students and maintain cleanliness around the school premises, including drains. (P7, F, Head Teacher)*

#### 3.6.4 Traditional remedies

Traditional approaches such as the use of the Tulasi plant, reflect cultural practices in the malaria prevention strategies in schools.

*We planted ‘Tulasi’ (Ocimum tenuiflorum) to tackle malaria in our school. We have advised students to consume one leaf daily on an empty stomach. It will prevent malaria attacks. (P11, M, Head Teacher)*

#### 3.6.5 Malaria screening

Regular screening of malaria among students is used as a preventive strategy against malaria in some schools. This is often been done with the collaboration of health authorities such as primary health centers (PHC).

*In our school, regular health check-ups are conducted by the health department. (Birida)*

*We conduct check-ups for malaria in our school hostel. All students, including teachers, have received mosquito nets. (P 10, M, Head Teacher)*

*We regularly collaborate with local health authorities and CHC (Community Health Centre) people for malaria medication and vector control measures. (P18, M, Head Teacher)*

### 3.7 Responsive strategies

This section analyses malaria-responsive strategies implemented by schools to address malaria cases among students in the study area.

#### 3.7.1 Diagnostic testing

Testing for malaria is an important responsive strategy employed by many schools, as narrated by the respondents. Initially, when students with fever symptoms, immediate attention is given, typically starting with treatment using paracetamol. However, if the fever persists or worsens, schools proceed to conduct a blood test with the help of ASHA workers to confirm malaria. This allows for early detection and treatment of cases among students.

*We administer paracetamol initially and conduct blood tests if the fever persists, providing prescribed medicines thereafter. (P24, M, Teacher)*

*In our school, when a student complains of a fever, we initially treat it as a common cold. If the fever persists despite paracetamol, we conduct a blood test to confirm malaria. (P4, M, Teacher)*

#### 3.7.2 On-site treatment and referrals

Many schools have policies that allow malaria testing and treatment on school premises with the help of ASHA workers, ensuring prompt care unless serious complications arise. However, some participants reported taking students to nearby health facilities for testing and treatment.

*According to our school policy, we provide treatment on-site unless a serious complication arises. (P2, F, Teacher)*

*We provide treatment for students within our school premises. Recently, a sixth-grade student received a 14-day treatment entirely at our school. We did not send him home. (P1, F, Hostel Warden)*

*We provide treatment within the school premises, and if necessary, we refer students to the nearby Community Health Centre (CHC). (P17, F, Teacher)*

#### 3.7.3 Sick room

Many schools maintain a sick room equipped with mosquito nets for students diagnosed with malaria, ensuring they receive special attention until they fully recover from the disease.

*We have a sick room in our hostel. There, we keep them under mosquito nets and provide special attention until they fully recover. (P24, M, Teacher)*

*We do not allow students to go home during treatment days to ensure they complete their medication course. (P7, F, Head Teacher)*

### 3.8 Malaria and school absenteeism

While most of them acknowledge past challenges, they generally agree that malaria’s influence on attendance has lessened due to improved access to medication and on-site treatment. Although sporadic cases of absenteeism due to malaria still arise, they are promptly managed.

*In the past, we used to face issues with absenteeism due to malaria in this school, especially during the rainy season. However, now we have an ample supply of medicines, and absenteeism has decreased considerably.*

*…at present, malaria does not have a significant impact on students’ attendance, as treatment is readily available within our school premises. However, in the past, students missed classes due to malaria.*

*I have not noticed much impact on attendance since affected students are promptly treated. However, there have been occasional instances, such as some students being absent for three to four days due to malaria.*

However, participants underscore logistical hurdles during the rainy season, such as waterlogging and transportation issues, which affect student’s attendance. One participant cited a 20–30% reduction in attendance during rainy seasons due to logistical hurdles faced by students, from areas cut off.

*During the rainy season, there is a 20–30% reduction in attendance, primarily due to logistical challenges faced by students from areas cut off. However, this decrease is not solely attributed to malaria but also due to obstacles like waterlogging. (P17, F, Teacher)*

*There is a 10–20% drop in attendance during the rainy season. These students come from isolated areas without access to communication. (P22, F, Head Teacher)*

### 3.9 Perceived challenges

This section sheds light on challenges regarding malaria in schools and possible solutions recommended by the participants in this study.

#### 3.9.1 Trained teachers

Many schools are facing challenges due to the absence of an adequate number of trained teachers who are capable of managing malaria patients, as stated by participants. As many of these schools in the study area are located in remote areas, with a majority of students hailing from disadvantaged communities, they lack easy access to healthcare facilities. They noted, that equipping teachers in malaria management can reduce the need for patients to travel to distant health centers.

*I think all residential schools should have trained teachers equipped to provide immediate treatment to students. Also, access to testing kits within schools would enable us to undertake prompt diagnosis and treatment. We may not need to travel long distances for the same. (P4, M, Teacher)*

#### 3.9.2 Insecticide-treated nets

Several participants highlighted the supply issues of insecticide-treated nets. Many nets in hostels were damaged or unusable due to the lack of consistent distribution. However, participants noted that the state education department distributed ordinary mosquito nets without insecticide treatment recently.

*We received insecticide-treated nets in 2017. After that, we did not receive any more insecticide-treated nets. (P1, F, Hostel Warden)*

*In our school, many nets are damaged or unusable, and no nets have been distributed since 2017. Recently, our department provided ordinary mosquito nets. (P7, F, Head Teacher)*

#### 3.9.3 Dedicated Auxiliary Nurse Midwives

Challenges in accessing healthcare personnel, such as Auxiliary Nurse Midwives (ANMs), as cited by participants. They stated that the distance between schools and healthcare facilities, coupled with inadequate staffing, results in insufficient healthcare provision.

*We request a dedicated ANM for our residential school, as the current ANM oversees five schools, making it difficult for her to attend to our needs promptly. She is staying 18 kilometres away from here (P10, M, Head Teacher)*

*If a permanent ANM were assigned to our school, it would greatly benefit us. Currently, there is only one ANM responsible for overseeing eight schools in our area. She visits our school twice a month. (P16, F, Hostel Warden)*

#### 3.9.4 Traditional healing practices

Traditional beliefs often influence treatment-seeking behavior among school children, leading to delays in proper medical intervention. As traditional healing practices are prevalent among tribal communities in Odisha, many parents opt for alternative treatments from traditional healers, which may not be effective for malaria. Participants in this regard state:

*…I observed that traditional beliefs, such as seeking treatment from village healers, can also delay proper medical intervention. (P17, F, Teacher)*

*We do not leave them in their homes because their parents will not take proper care of them. They may go to the ‘Adivasi gunia’- a traditional healer for herbal treatment instead of Chloroquine. One student’s father forcibly took his son to be treated in his home. But when I came to know that the patient’s condition deteriorated, I went to his home and brought the patient to the school for treatment. (P23, M, Head Teacher)*

#### 3.9.5 Shortage of medicines

The participants, mostly from residential schools highlighted medicine shortages, compromising their ability to effectively treat malaria cases in schools.

*Our department is unable to supply sufficient medicine. ACT (Artemisinin combination therapy) supply is suboptimal. In our school, while doing mass tests, they are not able to give ACT to all of the Pf-positive cases. They tell us to adjust. (P23, M, Head Teacher)*

*The hospital is 14 kilometres away from our school. If there is no medicine at CHC we have to go to Singhpur, which is 30 kilometres from here. So, we are always facing medicine shortages. (P25, F, Head Teacher)*

#### 3.9.6 School vacations

School children returning from home after vacations often come back with malaria, as reported by teachers. This occurrence may be attributed to a lack of safe housing facilities, inadequate awareness among parents and lack of usage of preventive measures at villages.

*I have observed that, whenever they go home during vacation, they return to school with malaria fever. I think their parents are not aware of malaria. (P22, F, Head Teacher)*

#### 3.9.7 Migration to “Kudias”

Participants reported that many families migrate to temporary shelters known as “*kudias*” (small huts inside forested areas), especially during harvesting seasons. Since most *kudias* lack basic facilities, children are exposed to the risk of malaria.

*I think the migration of the villagers to ‘kudia’ should be restricted or otherwise, they should be encouraged to use nets at kudias’. (P24, M, Teacher)*

#### 3.9.8 Poverty and lack of risk perception

The poor financial situations of parents impact the health priorities of their children. Most of the children belong to the tribal communities and they are financially poor due to historical reasons. Often, their focus on survival overrides their concern for the health and well-being of their children due to economic constraints.

*…because of their poor financial situation, tribal communities do not take their health seriously; instead, they are more concerned with day-to-day survival rather than well-being. (P12, M, Head Teacher)*

## 4 Discussion

This study offers insights into the influence of malaria on school absenteeism, shedding light on local strategies for managing the disease among primary school children and the ongoing challenges in an endemic region of India. Despite the continued presence of malaria, its influence on absenteeism appears to be less significant than previously assumed. While prior research established a direct link between malaria and school absenteeism (12, 28), this study found a relatively stable absenteeism rate (≥30%) across periods of both high and low malaria endemicity.

Several factors may explain this unexpected finding. First, the risk of *P. falciparum* malaria among children aged 6–14 was reported lower compared to other age groups (29), likely reducing its impact on school attendance. Second, the implementation of various anti-malaria programs by the local government, such as the Odisha Health Sector Plan (OHSP), Comprehensive Case Management Project (CCMP), and Durgama Anchalare Malaria Nirakaran (DAMaN), has likely contributed to reduced malaria burden (30). Third, the mid-day meal scheme, providing free lunch to students, may artificially inflate attendance rates, especially in rural and deprived areas (31). Finally, the high prevalence of asymptomatic malaria among school-aged children may lead to an underestimation of its effect on school attendance, as asymptomatic cases often go undetected (32, 33).

The schools included in this study were predominantly *Ashram* schools, specialized institutions administered by the state government in tribal and remote regions. An evaluation study of Ashram schools in 2019 revealed an absenteeism rate of 3 to 8 days annually, aligning with the lower rates observed in this study (34). Family illnesses, delayed returns after vacations, and local festivals were identified as the primary reasons for absenteeism. While qualitative interviews in this study indicated a diminishing trend of malaria-related absenteeism, logistical hurdles during the rainy season continue to be a significant contributing factor, aligning with findings from previous research (35).

Significant variation in absenteeism rates was observed among different schools, ranging from 1.3 to 3.6%, suggesting the influence of local contextual factors. Previous research has highlighted the role of socioeconomic conditions, distance to schools, educational infrastructure, weather patterns, health issues, and cultural beliefs in shaping school absenteeism in India (36–38).

Teacher participants in this study noted a substantial reduction in malaria cases among primary school children over the years, consistent with the overall decline in malaria in India (1, 20). However, seasonal fluctuations in malaria cases persisted, emphasizing the disease’s complexity and the need for continued vigilance. The decline in malaria among school children is attributed to various interventions including the widespread adoption of LLINs in residential schools, health education initiatives, enhanced access to malaria testing and treatment within schools and intensified vector control efforts. These findings align with existing studies highlighting the effectiveness of such interventions in controlling malaria (39–41).

We found that a range of strategies were implemented in schools to prevent malaria transmission and manage infected students. These strategies encompass health education, vector control, promotion of LLIN among hostel-dwelling students, traditional remedies, malaria screening, testing, on-site treatment, and the provision of sick rooms. These measures offer valuable insights that can be replicated in similar settings to combat malaria. Previous research consistently supports the effectiveness of such school-based malaria control efforts (42–44), given the school’s unique access to children in the school-age group (15).

Most schools in our study focused on health education activities and the distribution of mosquito nets, both of which are widely recognized as effective tools in curbing malaria (14, 45, 46). Studies from Africa have shown that the use of mosquito nets by school-age children significantly lowers the risk of *P*. *falciparum* infection (18, 19). In Odisha, over 90% of Ashram schools maintain a mosquito net register, reflecting their commitment to malaria prevention (34).

On-site treatment of malaria within school premises found another effective strategy in this study, as demonstrated by studies in Ghana, Malawi, and Tanzania (47–50). Establishing sick rooms equipped with mosquito nets for malaria-infected students was another proactive approach that aligns with the effectiveness of case isolation seen during the COVID-19 pandemic (51). This initiative is particularly valuable in resource-constrained settings. Additionally, incorporating traditional practices like using the *Tulasi* plant reflects cultural influences on malaria prevention, especially in tribal communities. Previous studies have identified over 16 traditional plant species used for prophylactic purposes against malaria in Odisha (52, 53).

Challenges however persist, including a shortage of trained teachers, medical supplies, and healthcare infrastructure in remote and tribal areas (54). While the government’s initiatives to empower Accredited Social Health Activists (ASHA) in malaria management since 2010 have alleviated some of these issues (55), concerns remain regarding the inadequate supply of insecticide-treated nets and the reliance on untreated nets.

Seasonal migration to temporary shelters known as *“kudias”* and reliance on traditional healing practices can increase the risk of malaria, necessitating the need for community education and promotion of net usage, especially in migratory settings. We found that school vacations also facilitate malaria transmission, as returning students may reintroduce the disease from their villages. To effectively address this, comprehensive strategies are necessary, including parental education and community-level interventions. Additionally, poverty and low-risk perception among marginalized Indigenous communities pose significant challenges, as health priorities often take a backseat to economic concerns. Addressing these issues requires tailored interventions, community engagement and socio-economic development in disadvantaged areas, in line with WHO recommendations (56).

### 4.1 Toward understanding malaria and school attendance: conceptual framework

**Figure 5:**
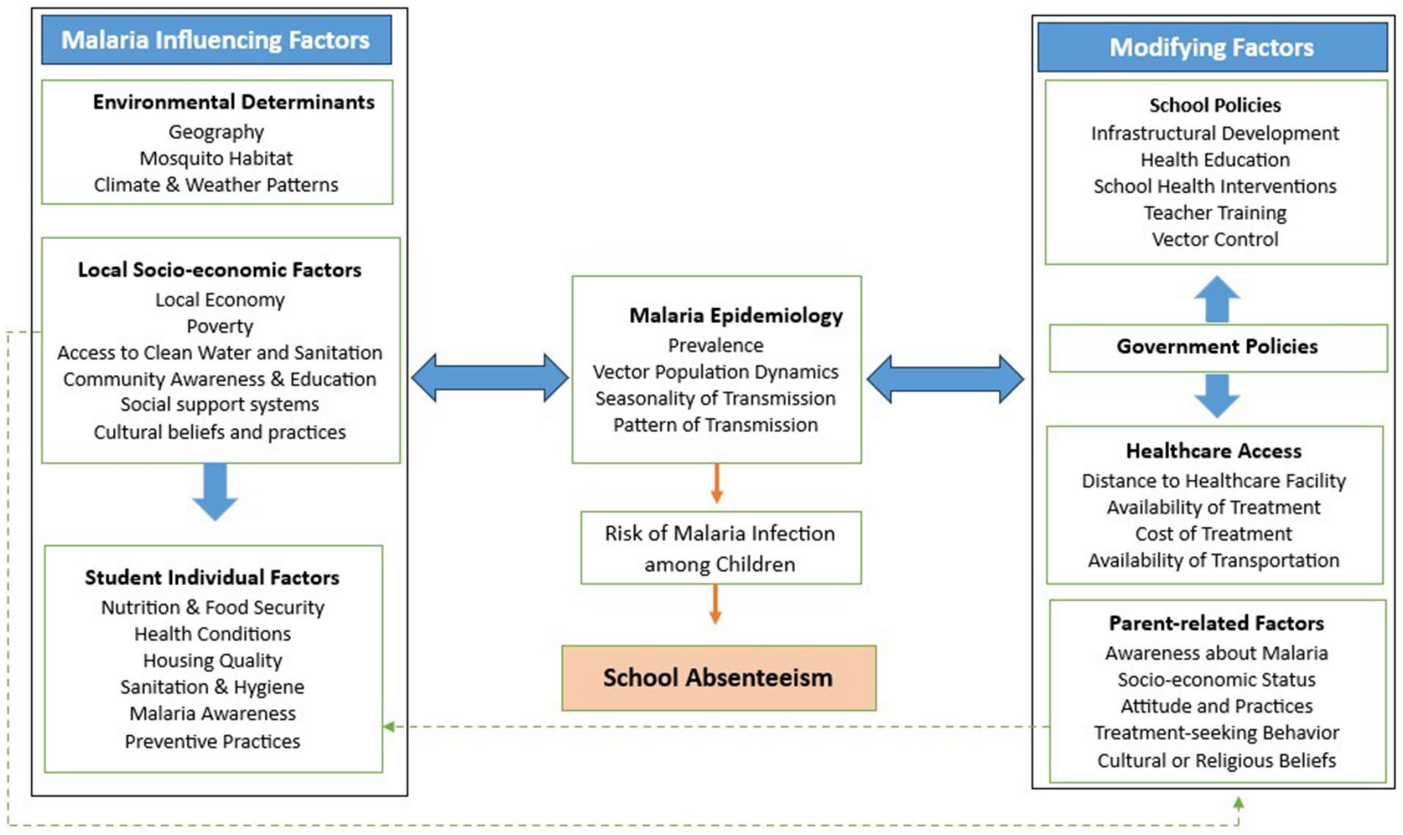
Conceptual framework for malaria and school absenteeism.

Based on the study results, we developed a conceptual framework elucidating the interplay between malaria and school absenteeism among school-aged children (Figure 5). Environmental factors, such as geography, mosquito habitat, and climate patterns interact with local socio-economic conditions, including poverty, access to clean water and sanitation, and cultural beliefs and practices, thereby shaping malaria risk. Additionally, individual factors such as a child’s nutritional status, food security, health condition, housing quality, sanitation and hygiene and preventive practices contribute to their susceptibility to malaria. These factors collectively influence malaria epidemiology, including its prevalence, vector dynamics, and transmission patterns, which in turn affect infection rates among children. The framework further recognizes the importance of modifying factors, such as school policies (infrastructure development, health education and health interventions), government interventions and healthcare access (availability, cost and transportation facilities), in mitigating the impact of malaria on school absenteeism. Parent-related factors, including socio-economic status, awareness, cultural beliefs, and treatment-seeking behavior, also play a significant role in shaping children’s malaria risk and school attendance. These modifying factors either increase or reduce malaria risk among school children depending on their implementation or accessibility. Ultimately, these interrelated factors interact to influence malaria prevalence and, consequently school absenteeism.

### 4.2 Strengths and limitations

The key strength of this study lies in its utilization of a mixed-methods approach. While quantitative data provided a broad overview of absenteeism trends and patterns across academic years and regions, the qualitative data gathered through teacher interviews shed light on the underlying factors influencing malaria-related school absenteeism and the local strategies employed to address them. This combination of methods offered a more holistic understanding of nuanced issues, such as the role of local cultural practices and seasonal migration—insights that would not have been captured through either method alone. This application of mixed methods can significantly enrich our understanding of context-specific health issues like malaria, where local factors play a crucial role in the effectiveness of interventions. However, the study’s findings may be subject to several limitations. First, the reliance on manual attendance registers may introduce data collection errors (27). Focusing solely on Ashram schools may limit the generalizability of results. Qualitative data can be subject to biases. Additionally, geographical and seasonal factors, resource constraints, and cultural practices may confound the results. Importantly, the study did not evaluate the effectiveness of the implemented preventive measures in reducing absenteeism.

## 5 Conclusion

The study explored the influence of malaria on school absenteeism in Odisha. While malaria cases have largely declined due to various interventions in the state, absenteeism rates remained relatively stable, suggesting that factors beyond malaria play a role in school attendance. Schools have adopted a range of proactive strategies to combat malaria, actively engaging in health education, preventive measures and case management. However, ongoing challenges, such as a shortage of trained staff, medical supplies and limited healthcare infrastructure, particularly in tribal and remote regions, require further attention. Future studies could investigate the long-term effects of malaria interventions on both absenteeism and academic performance, with a focus on implementation research to evaluate the effectiveness of school-based malaria case management programs. Quantitative methods can further explore the adoption and effectiveness of malaria preventive measures in schools.

## Data Availability

The original contributions presented in the study are included in the article/supplementary material, further inquiries can be directed to the corresponding author.
